# Filling in the gaps: evidence of leaf endodermis and vein sheath in gymnosperms

**DOI:** 10.1093/aob/mcaf165

**Published:** 2025-07-19

**Authors:** James L Seago, Kamal I Mohamed, Kristen R Haynes

**Affiliations:** Department of Biological Sciences, SUNY Oswego, Oswego, NY 13126, USA; Department of Biological Sciences, SUNY Oswego, Oswego, NY 13126, USA; Rice Creek Field Station, SUNY Oswego, Oswego, NY 13126, USA; Rice Creek Field Station, SUNY Oswego, Oswego, NY 13126, USA

**Keywords:** Endodermis, Casparian bands or strips, conifers, gymnosperms, needles, phylloclades, vein sheath

## Abstract

**Background and Aims:**

Botanical literature is filled with studies which tried to demonstrate that leaves of many gymnosperms have an endodermis with Casparian bands or a sheath of sclerified cells around leaf veins. Direct photographic evidence of an endodermis with Casparian bands (strips) is lacking for the leaves of many gymnosperms. Our goal was to confirm with direct evidence an endodermis with Casparian bands or a vein sheath in leaves of representative gymnosperms via histochemical staining and epifluorescence microscopy, while extending previous work by examining understudied petioles and leaf bases.

**Methods:**

We sectioned leaves fresh with razor blades and viewed them unstained or stained, usually with berberine hemisulfate, counterstained with gentian violet, and phloroglucinol HCl. Bright-field, epifluorescence and/or laser confocal microscopies on a Zeiss LSM700 were used for viewing and imaging.

**Key Results:**

Most members of Pinaceae of Pinophyta (13 species of genera *Cedrus, Pseudotsuga*, *Larix*, *Picea* and *Pinus*) had endodermis with distinct Casparian bands in needles. We identified endodermis in the distal petiolar regions of nine of these same species. Sclerified vein sheaths or partial vein sheaths were observed in 10 species of 41 studied among Cycadophyta (*Cycas*), Ginkgophyta (*Ginkgo*) and the Pinophyta Podocarpaceae (*Dacrycarpus*, *Dacrydium*), Cupressaceae (*Metasequoia*), Taxaceae (*Amentotaxus*) and Pinaceae (*Keteleeria*, *Abies, Tsuga*).

**Conclusions:**

Endodermis with Casparian bands is only characteristic of most species of Pinaceae; vein sheaths are found in three genera of Pinaceae and six genera of the other families of gymnosperms, but most gymnospermous leaves lack endodermis and vein sheath, particularly in petiolar and leaf base regions. The presence of endodermis with Casparian bands could have contributed to the adaptation of the Pinaceae to extreme environments; members of genera such as *Picea* and *Pinus* are the typical treeline species in many mountain ranges across the world.

## INTRODUCTION

The leaves of gymnosperms have been variously described as linear with abaxially/adaxially oriented veins, broad with petioles, needles with petioles, cataphylls or scale leaves, cladodes with obliquely oriented veins, or phylloclades with perpendicularly orientated veins and stem-like concentrically arranged vascular bundles (Napp-Zinn, 1966; Bierhorst, 1971; Gifford and Foster, 1989; Dörken and Stützel, 2011; Kaplan, 2022). Within these leaves, structures surrounding the vascular tissues, or veins, have been described and designated in many different ways, but usually structures termed vascular bundle sheaths (vein sheaths) or endodermis have been assigned to them; both of these are historically known.

From Chamberlain (1935) to Napp-Zinn (1966), Esau (1977), Gifford and Foster (1989), Biswas and Johri (1997) and Kaplan (2022), texts and major books have long extolled the characteristics of gymnosperm leaves, including the occurrence of endodermis, especially in leaves of pines and related Pinaceae. In his review less than 30 years ago, however, Lersten (1997) noted that there was no real proof of Casparian bands in an endodermis in gymnosperm leaves, and earlier, in his major tome on gymnosperm leaves, Napp-Zinn (1966, citing mostly much earlier researchers) did not present any photographic evidence of leaf endodermis or of sclerified vein or vascular bundle sheath (or ‘Leitbündelscheiden’, pp. 156–157, 226–227). More recently, the Dörken group has studied many of the families, genera and species of gymnosperms (e.g. Dörken *et al*., 2010, 2019, 2021, 2023*a*, *b*; Dörken and Stützel, 2011, 2012; Dörken, 2012, 2013*a*, *b*, 2014; Dörken and Parsons, 2016; Dörken and Lepetit, 2018; Dörken and Nimsch, 2018, 2021), but their techniques did not usually allow direct Casparian band evidence.

Endodermis and vein sheaths are different anatomical structures, although both form the innermost layer of root and shoot cortex or leaf mesophyll, and will be illustrated initially in the first figure of the Results section. The endodermis in vascular plants is a specific structure that has been most characterized in roots. In its simplest form, as described and discussed variously by Peterson and colleagues (Seago *et al*., 1999; Enstone *et al*., 2003; Ma and Peterson, 2003), Schreiber and Franke (2011) and Soukup and Tylová (2018), it comprises a ring or layer of cells of the innermost cortex with mostly lignified Casparian bands impregnated in its radial and transverse cell walls: a State I endodermis has Casparian bands only. Endodermis can also form additional chemical structures; a State II endodermis has Casparian bands with associated suberin lamellae within the limits of the primary cell wall and outside the protoplast. Further, endodermis can form a State III endodermis with secondarily lignified wall lining the inside of the suberin lamellae, often with its thickness greatest on the inner tangential wall in root endodermis (and outer tangential wall of an innermost layer of an exodermis, which may form the outermost layer of cortex). An endodermis in stems has also been well documented (Seago, 2020), as has endodermis of some gymnosperm roots (e.g. Wilcox, 1968 for *Pinus resinosa*; Bonacorsi and Seago, 2016 for *Ginkgo biloba*; Song *et al*., 2019 for *Cunninghamia lanceolata*; Brundrett and Tedersoo, 2020 for various gymnosperms with mycorrhizae).

Importantly, as noted by Enstone *et al*. (2003), Geldner (2013), Soukup and Tylová (2018) and others, the endodermis regulates the movement of water, dissolved minerals and carbohydrates into and out of the vascular cylinder of roots; further, the endodermis filters and arrests unwanted harmful molecules from being translocated in the xylem strand up to the shoot while at the same time allowing necessary inorganic molecules essential for plant growth. While water and dissolved minerals can pass apoplastically from cell wall to cell wall, symplastically or even transcellularly through the protoplasts of cortex cells, all movement of water and minerals passes through the endodermis only symplastically or transcellularly due to the presence of Casparian bands in the radial/transverse walls of the endodermis. It is known that in the needles of conifers, endodermis also plays an important ecological role in the survival of pines in extreme cold habitats; for example, Stegner *et al*. (2023) suggested that the lignification of the endodermis blocks the penetration of ice from the frozen vascular tissue into the mesophyll, allowing needles to remain active during extended periods of extreme cold temperatures. This, in turn, may be associated with oxygen and carbon dioxide pathways between vein and mesophyll (Roden *et al*., 2015; Sage and Khoshravesh, 2016) as well as movement of nutrients like nitrogen (Gao *et al*., 2023; Valderrama-Martin *et al*., 2024) and carbohydrate (Liesche *et al*., 2011) and structural support (e.g. Leegood, 2007; Meicenheimer *et al*., 2008).

An endodermis with Casparian bands has been well demonstrated in leaf blades or needles in relatively few species, with the most striking examples being in *Pinus nigra* and *P. resinosa* of the Pinaceae by Meicenheimer *et al*. (2008), where *P. nigra* had prominent Casparian bands and *P. resinosa* had prominent outer tangential, secondarily lignified walls in its endodermis. Other authors (e.g. Campbell, 1972; Delian and Săvulescu, 2022) have presented photographs of *P. nigra* clearly showing endodermis, sometimes with well-demonstrated Casparian bands. Often published illustrations are specimens in which Casparian bands do not show, but the cells have a characteristic endodermal shape and pattern, as in Esau (1977) for *Larix* and *Pinus* and Gifford and Foster (1989) for *Pinus*. Using a variety of techniques, Soar (1922) had drawings of leaf endodermis in *Pinus*, *Picea*, *Abies*, *Larix*, *Pseudotsuga*, *Cedrus* and *Tsuga*. Others (e.g. Hu and Wang, 1984; Eguchi *et al*., 2004; Bercu *et al*., 2010; Ghimire *et al*., 2015*a*) demonstrated images with endodermis patterns in a variety of Pinaceae, but Casparian bands were not visible. Vein sheath was labelled in *Abies* (Dörken, 2014; Dörken and Lepetit, 2018); these studies were not suggestive of an endodermis with Casparian bands.

Evidence seems to show that endodermis in gymnosperm leaves may be a more recently derived trait and of interest evolutionarily. Fossil cycads, for example, do not have endodermis in cataphylls or scale leaves (Hermsen *et al*., 2006), and they may be among the oldest of gymnosperms (e.g. Taylor *et al*., 2009; Crisp and Cook, 2011; Lu *et al*., 2014; Lubna *et al*., 2021). Fossils from basal gymnosperms like Podocarpaceae (Andruchow-Colombo *et al*., 2023) do not show any evidence of endodermis in leaves. On the other hand, fossil pines, like *Pinus hokkaidoensis* (Stockey and Ueda, 1986) and *P. arnoldii* (Klymiuk *et al*., 2011), are recent in evolutionary appearance and have been shown to have a distinct endodermis (Taylor *et al*., 2009; Gernandt *et al*., 2016).

A vein sheath or its modifications consists of cortex sclerenchyma cells surrounding the vascular bundles that are sclerified or lignified without Casparian bands. The occurrence and nature of vein sheaths in gymnosperm leaves have been little addressed in major tomes (e.g. Bierhorst, 1971; Gifford and Foster, 1989; Kaplan, 2022), but they have been variously described as a ‘layer of lignified cells’ in *Ginkgo* by Dörken (2013*b*, p. 90), a ‘parenchyma sheath’ or ‘unstratified endodermis with thin-walled large cells’ by Cargo *et al*. (2000, p. 38), or a zone of ‘scattered thin-walled lignified fibers’ in *Cycas* by Griffith *et al*. (2014, p. 241). Vein sheaths are clearly irregular in pattern and structure, but without Casparian bands. In addition to facilitating the movement of water, nutrients, photosynthates and gases between vascular tissues and mesophyll, the vein sheath reinforces and supports the vascular tissues and even the inner mesophyll (Esau, 1977; Leegood, 2007), potentially serving a similar function as endodermis.

Among other gymnospermous families, evidence of endodermis with Casparian bands or vein sheath is spotty and sometimes lacking. Sethi *et al*. (2020) reported a sclerenchyma sheath in *Cycas*. Cargo *et al*. (2000, p. 38) stated ‘an unistratified endodermis with thin-walled large cells’ in *Ginkgo biloba*, but it was not so described by Duarte *et al*. (2013); Dörken’s (2013*a*) bright-field micrographs demonstrated a vein sheath in its lamina. Liesche *et al*. (2011, p. 184) reported a ‘suberized bundle sheath’ and ‘endodermis-like bundle sheath’ for the same lamina image in *Ginkgo*, whereas Klimko *et al*. (2015, p. 185) noted a ‘parenchymatous sheath’ around its veins. Bundle sheaths appear to be present in some members of the Podocarpaceae (Andruchow-Colombo *et al*., 2024) and not others (Lee, 1952; Griffith, 1957). Endodermis is claimed but not demonstrated in leaves of Cupressaceae by Yousif and Shahbaz (2023); presumed endodermis was also noted in *Juniperus* (Bercu *et al*., 2010; Güvenc *et al*., 2011). In their broad survey of leaf characteristics in conifers (7 families, 37 genera, 111 species), Yao and Hu (1982) reported the presence or absence of endodermis or vein sheath, but their drawings did not demonstrate endodermis with Casparian bands or vein sheaths. The scale leaves or cataphylls reported by many (e.g. Soar, 1922; Sacher, 1955; Kaplan, 2022) have reduced vascular tissue and no apparent endodermis.

Thus, the evidence for endodermis with Casparian bands in gymnosperm leaves is incomplete and uncertain. The situation in petioles or leaf bases has been far less documented, even though Napp-Zinn (1966), for example, devoted much to leaf origin – without a single depiction of a mature leaf with petiole. Lee (1952, p. 395) illustrated by drawing a petiole and its leaf vascular bundle without an ‘endodermal sheath’ in the leaf of *Dacrydium taxoides* of the Podocarpaceae. An illustration by Cross (1940) showed a vein sheath in the petiolar or proximal region of a *Cunninghamia lanceolata* leaf. Doak (1935) had only drawings of the fascicle bases in pine and leaf bases in Araucariaceae. Soar’s (1922) drawings of the origin of needles in fascicles of *Pinus sylvestris* showed no endodermis in lower or proximal levels of the developing needles and an incomplete endodermis in *Picea excelsa* and in *Abies pectinata*. Needles of *Pinus mugo* and *P. monophylla* were photographed by Bierhorst (1971) with endodermis around the two vascular bundles of a needle; his photograph of a *P. mugo* fascicle without endodermis is one of the few examples of photographic evidence of endodermis presence or absence in petiolar regions within a fascicle of gymnosperm leaves. Bright-field images of *Picea mariana* needles by Way and Sage (2008) show a typical endodermis but without manifest Casparian bands. Hu and Yao (1981) and Hu and Wang (1984) reported an endodermis in the needle and stem of *Cathaya argyrophylla*; an endodermis is apparent in its leaves without visible Casparian bands and in a petiole in a ring around the vein, but endodermis or vein sheath was not mentioned in its petiole. Duarte *et al*. (2013) had micrographs of petioles in *Ginkgo biloba* without noting vein sheath or endodermis, but Dörken (2013*a*) clearly illustrated one vein sheath around the two vascular bundles in its petioles. Yet Klimko *et al*. (2015) labelled an endodermis in *Ginkgo* petioles. The dearth of photographic evidence and conflicting reports indicate that more investigations are merited for petioles in gymnosperms.

Given the paucity of photographs adequately demonstrating an endodermis with Casparian bands or vein sheaths in gymnosperm leaves, we examined leaves (the Dörken group; Napp-Zinn, 1966; Kaplan, 2022) and other gymnospermous leafy appendages like cladodes (Dörken and Stützel, 2011), phylloclades (Dörken *et al*., 2021; Kaplan, 2022), cataphylls (Kaplan, 2022) and their associated leaf petioles or petiolar regions (or leaf bases where no petiole was apparent), which have been rarely included in analyses of leaves. We demonstrate the presence or absence of endodermis with Casparian bands along radial (and sometimes transverse) cell walls, mostly by epifluorescence of magnified images after selective staining and microscopy. We concentrate on the endodermis and the xylem of the vascular tissue because xylem is much easier to demonstrate in relation to Casparian bands than is phloem tissue. For many species, we use higher-magnification close-ups of veins to show more clearly the presence or absence of endodermis Casparian bands. We will note other leaf features, like transfusion tissues, which may be appropriate to anatomical characterizations and functions in veins. It is sometimes very difficult to determine a vein sheath with lignified cell walls but no Casparian bands. These observations should help researchers studying the associations among plant structure and function in various ecological/environmental conditions. We have at least one image for all species studied so that we can demonstrate the situations in many species and genera, but we emphasize, with more detail, select species among 11 families, 30 genera and 41 species.

## MATERIALS AND METHODS

For two centuries, Oswego, NY, USA, has been a small but major port on Lake Ontario, easternmost of the North American Great Lakes that flow into the St Lawrence River and then the Atlantic Ocean. During that time, ships have brought seeds or plants into the Oswego area from many places around the world; for example, on the SUNY Oswego campus there are non-native species of gymnosperms, including dawn redwood or *Metasequoia glyptostroboides*, which is believed to have been planted on campus in the 1950s, not many years after it was reported extant in China, and *Chamaecyparis pisifera*, located within 50 m of Lake Ontario shoreline. In the city of Oswego, well-established trees of *Ginkgo biloba*, *Cryptomeria japonica*, and *Picea abies* occur within 200 m of Lake Ontario and the Port of Oswego, and in the surrounding towns of Minetto, Oswego and Scriba many other non-native as well as native gymnosperms are found.

With that background, we were long interested in these gymnosperms. Leaves were obtained from plants growing in the city of Oswego, NY, USA, the towns of Minetto, Oswego and Scriba, NY, USA, the campus and greenhouse of SUNY at Oswego, and a few botanical gardens and growers (examples are named in the Acknowledgements section). Multiple leaves of deciduous species like *Ginkgo biloba* and *Larix occidentalis* were sampled during early to late summers, June into October. Evergreen species like *Chamaecyparis pisifera* and *Pinus strobus* were sampled during their first year from early summer into winter, and we examined leaves in summer and winter (we note, however, that summer to winter first-year leaves were similar in structure). Leaves and stems were fresh-sectioned with Wilkinson sword razor blades (split in half to make single blades; sometimes the halves were held together to section through a leaf, a technique taught to J.L.S. by Chaodong Yang). Specimens were then run through stain procedures or mounted directly in dH_2_0 or 80 % glycerine.

Following methods in Seago *et al*. (1999), Soukup and Tylová (2018) and Seago (2020), staining procedures of freshly cut specimens included berberine hemisulfate for 1–2 h to reveal leaf cell walls under epifluorescence, berberine hemisulfate counterstained briefly in 1 % gentian violet or in 0.5 % toluidine blue O for 30–60 s to reveal lignins in endodermis Casparian bands and xylem under epifluorescence, fluorol yellow for 1 h to reveal suberin under epifluorescence, phloroglucinol HCl for 5–10 min to stain lignin in cell walls in bright-field, Sudan red 7B or Sudan IV for 1 h to reveal suberin in bright-field, and toluidine blue O for 30–60 s as a metachromatic stain to colour different wall chemicals of various tissues in bright-field. These procedures allowed us to visualize endodermis with Casparian bands, vein sheaths, xylem tracheids and transfusion tissues (e.g. Hu and Yao, 1981). We do not include images of all of the staining methods.

We used a Zeiss LSM700 with a camera at SUNY Oswego for the vast majority of images under epifluorescence at 509 nm, but a few at 419 nm. Many Zeiss LSM700 images were taken in bright-field and in laser confocal microscopy at 488 nm, and an Amscope bright-field scope with a Samsung Galaxy A42 camera was used for some images.

## RESULTS

**Fig. 1.**
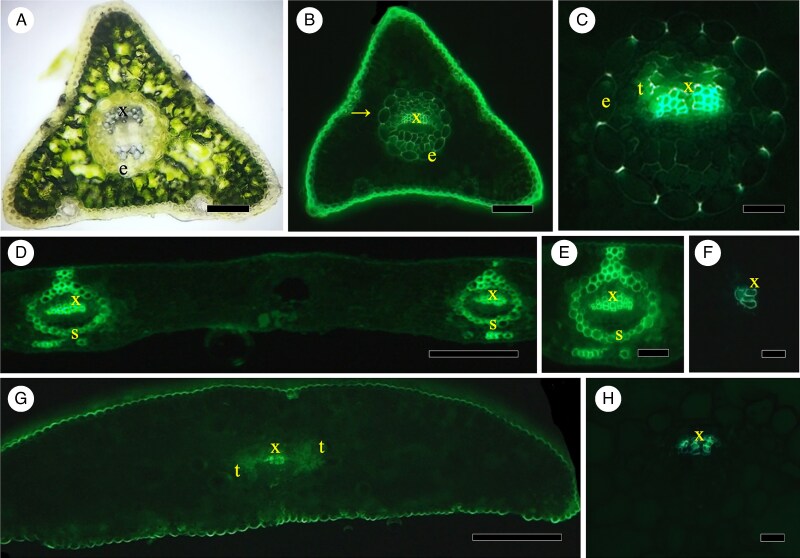
(A–C) *Pinus strobus*. (A) Transverse section of fresh, unstained needle, ring of endodermis without conspicuous Casparian bands, xylem of vein. Scale bar = 175 μm. (B) transverse section of needle stained in Bef (see abbreviations list at the end of the figure legend) and viewed in epifluorescence. Casparian bands easily visible, outer tangential cell walls of endodermis reveal some light lignin staining at arrow. Scale bar = 175 μm. (C) Close-up of BGVef-stained section with radially oriented Casparian bands of endodermis around xylem and some transfusion tissue. Scale bar = 60 μm. (D–F) *Ginkgo biloba*. (D) Transverse section of small section of laminar leaf with two large veins stained in Bef. Vein sheaths at s are around each vein, with xylem labelled. Scale bar = 400 μm. (E) Close-up of one such large vein with vein sheath, stained in berberine gentian violet and viewed in epifluorescence. Scale bar = 100 μm. (F) Small vein stained in BGVef viewed under epifluorescence does not reveal an endodermis with Casparian bands or vein sheath. Scale bar = 65 μm. (G, H) *Sequoia sempervirens*. (G) Transverse section of needle stained in Bef to show xylem of vein and transfusion tissue on either side of xylem, viewed in epifluorescence to show lack of endodermis and vein sheath. Scale bar = 350 μm. (H) Close-up of a vein stained in BGVef under epifluorescence to illustrate the lack of endodermis. Scale bar = 70 μm. Abbreviations in legends: bf, bright-field; ef, epifluorescence at 509 or 419 nm; lc, laser confocal at 488 nm; B, berberine hemisulfate; BGV, berberine/gentian violet; Pg, phloroglucinol 20 % HCl; Sr, Sudan red 7B; unst, unstained. Labels in photographs: c, concentric vascular bundles (stem-like); e, endodermis; s, vein sheath; t, transfusion tissue; v, vein; x, xylem.

After first presenting illustrations of specimens with endodermis, vein sheaths or no layers conspicuously surrounding veins (Fig. 1), the remainder of the Results will begin with the Cycadophyta, Ginkgophyta and Gnetophyta – gymnosperms not usually classified with the Pinophyta; these will then be followed by the Pinophyta from Araucariaceae through Pinaceae. These descriptions will include references to specific, appropriate literature.

### Structures surrounding leaf veins

Figure 1A is a transverse section of a fresh-cut, unstained bright-field image of a white pine (*Pinus strobus*) needle in which there is a clear ring of cells, i.e. endodermis, surrounding the central vein but no easily visible Casparian bands. Epifluorescence images reveal endodermis around a vein in Fig. 1B, C with the radially oriented Casparian bands showing clearly around the xylem tracheids and vein transfusion tissue. As seen in Fig. 1D, E, there is a vein sheath or ring or zone of lignified sclerenchyma cells around large veins in a small section of a broad maiden hair/ginkgo (*Ginkgo biloba*) leaf. Staining for Casparian bands in a small vein reveals that veins and vein sheath cells do not have Casparian bands (Fig. 1F). An example of a flattened gymnospermous needle in a coastal redwood (*Sequoia sempervirens*) without endodermis or vein sheath is shown in epifluorescence in Fig. 1G, H, where there are xylem tracheids and transfusion tissue visible in a vein, but no endodermal Casparian bands or sclerified cells.

### Cycadophyta, Cycadophyceae

#### Cycas revoluta

**Fig. 2.**
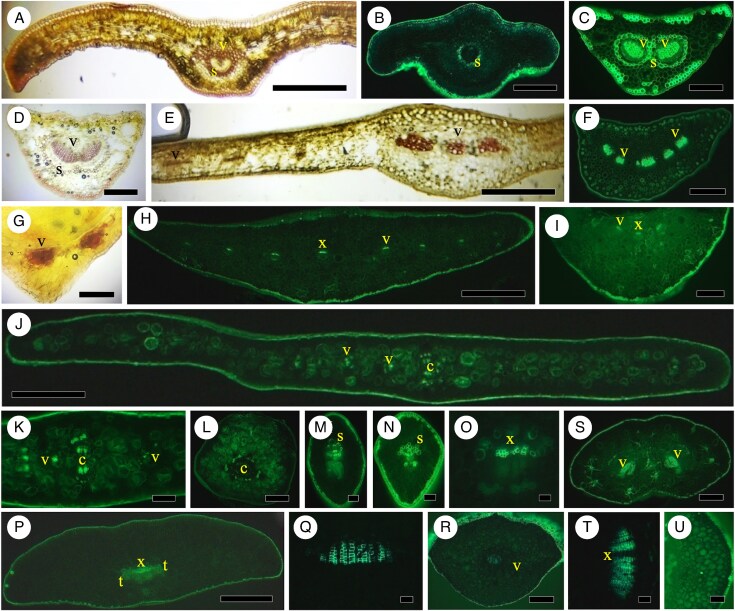
Specimens of Cycadophyta, Ginkgophyta, Gnetophyta and Pinophyta (Araucariaceae, Podocarpaceae, Sciadopityaceae). (A, B) *Cycas revoluta.* (A) Leaf section, vein with vein sheath, Pgbf (see abbreviations list in legend of Fig. 1). Scale bar = 300 μm. (B) Petiole, vein sheath, Bef. Scale bar = 150 μm. (C, D) *Ginkgo biloba* petiole (see also lamina in Fig. 1D–F). (C) Distal lamina, vein sheaths, Bef. Scale bar = 300 μm. (F) Proximal petiole near stem, one vein with two oblique halves, vein sheath, Pgbf. Scale bar = 300 μm. (E, F) *Gnetum gnemon*. Leaf blade, three main veins and lateral veins, no endodermis, no vein sheath, Pgbf. Scale bar = 400 μm. (F) Petiole, veins, two labelled, no endodermis, no vein sheath, Bef. Scale bar = 250 μm. (G) *Ephedra*. Scale leaves against stem, xylem cells are not revealed, Pgbf. Scale bar = 250 μm. (H, I) *Araucaria araucana*. (H) Leaf blade section with multiple veins and no endodermis or vein sheath, Bef. Scale bar = 500 μm. (I) Petiolar region at base of leaf, veins without endodermis or vein sheaths, Bef. Scale bar = 150 μm. (J–L) *Phyllocladus alpinus*. (J) Section of mid-phylloclade, c on concentric vascular bundles, v’s denote positions of some veins, Bef. Scale bar = 900 μm. (K) Phylloclade section of main concentric vascular bundles (two in number) and two veins labelled, no endodermis, Bef. Scale bar = 120 μm. (L) Concentric vascular bundles at phylloclade base, BGVef, no endodermis. Scale bar = 300 μm. (M) *Dacrycarpus dacrydioides*. Needle-like cataphyll, no endodermis, vein sheath cells, Bef. Scale bar = 100 μm. (N, O) *Dacrydium elatum*. (N) Cataphyll, vein sheath cells, Bef O. Scale bar = 100 μm. (O) Close-up of vein, no endodermis, vein sheath cells, BGVef. Scale bar = 60 μm. (P–R) *Podocarpus macrophyllus*. (P) Needle, vein with transfusion tissue, no endodermis, no vein sheath, Bef. Scale bar = 375 μm. (Q) Close-up of vein, no endodermis, BGVef. Scale bar = 90 μm. (R) Petiole, no endodermis or vein sheath, Bef. Scale bar = 270 μm. (S–U) *Sciadopitys verticillata*. (S) Transverse section of cladode, two obliquely oriented veins, Bef. Scale bar = 750 μm. (T) Close-up of one vein, xylem, no endodermis. Scale bar = 90 μm. (U) Scale leaf, no endodermis or vein sheath, little to no xylem. Scale bar = 150 μm.

The compound leaf of *Cycas revoluta* has leaflets (Fig. 2A) and a petiolar region (Fig. 2B) with a singular vein of little xylem surrounded by a vein sheath (Fig. 2B) but no endodermis. This is similar to the drawings in Lamb (1923) and to the photographs in Jeffrey (1917), Gifford and Foster (1989) and Stevenson *et al*. (1996). Griffith *et al*. (2014, p. 244) demonstrated a thick, U-shaped ‘limiting layer of vein’ with phloroglucinol stain, but no endodermis. The related *Zamia* leaves have many veins with only vein sheaths (Beck, 2010). Vovides *et al*. (2020) and Glos *et al*. (2022) show vein sheath very clearly in standard microscopic preparations in another cycad, *Ceratozamia*, and in *Zamia*, respectively, as well as Coiro *et al*. (2020) in various microscopies for many Zamiaceae.

### Ginkgophyta, Ginkgophyceae

#### Ginkgo biloba

The deciduous broad leaf lamina (Fig. 1D, E) and the petiole (Fig. 2C, D) of *G. biloba* long shoots and spur shoots (Gifford and Foster, 1989) are characterized by adaxial/abaxial vein orientation in the lamina with a vein sheath (Fig. 1D, E). There is no endodermis with Casparian bands around the leaf lamina or petiole veins as shown by berberine gentian violet staining in a small peripheral lamina vein (Fig. 1F). Along most of their length, petioles also have a vein sheath, extending even between the two veins of the petiole (Fig. 2C). Nearer petiole bases, xylem may be united and surrounded by a less distinct lignified vein sheath (Fig. 2D); there is no endodermis. This corroborates images by Dörken (2013*b*) and by Leigh *et al*. (2011) but contradicts the claims of an endodermis by Cargo *et al*. (2000) and Klimko *et al*. (2015, for petiole), neither of whom used techniques to demonstrate Casparian bands. Our *Ginkgo* leaves contrast with roots of *G. biloba*, which have been well documented (Bonacorsi and Seago, 2016) to have an endodermis and exodermis with Casparian bands and suberin lamellae, and even its first stem internode has such exodermis, which was revealed using similar techniques to the present study (from the same batch of seeds used to produce one of the plants in the present study).

### Gnetophyta, Gnetaceae

#### Gnetum gnemon

The broad leaf lamina (Fig. 2E) and narrower petiole (Fig. 2F) of *G. gnemon* are multi-veined with no endodermis (Fig. 2F) or vein sheath around the veins (Fig. 2E). Tomlinson and Fisher (2005) noted no endodermis but an ‘indistinct’ vein sheath in *Gnetum*, while Ajuru (2018) noted an endodermis, which does not appear evident.

### Ephedraceae

#### Ephedra tweedieana

Appressed to the stem, scale leaves (Fig. 2G) of *E. tweedieana* have no endodermis or vein sheath; these can also lack xylem in very small-scale leaves. Dörken (2012) showed no endodermis or vein sheath.

### Pinophyta, Araucariaceae

#### Araucaria araucana

The relatively wide leaf of *A. araucana* has several veins but no endodermis or vein sheath (Fig. 2H). Leaf tips reduce to one vein and leaf bases have a few veins with no endodermis (Fig. 2I). Thomson’s (1913) early photographs do not reveal a possible endodermis. Mastroberti and Mairath (2003) label endodermis in their images, but there is no clear ring of cells nor Casparian bands. The bright-field images of *A. araucana* by Rivera *et al*. (2022) or of *Wollemia* by Burrows and Bullock (1999) and Vasheka *et al*. (2019) do not mention or show endodermis or vein sheath. Multi-veined leaf and petiole images of *Agathis* do not reveal endodermis or vein sheath in Arbicheva and Pautov (2018).

### Podocarpaceae

Members of the Podocarpaceae have not been shown by most to have an endodermis around their vasculature structures in phylloclades, cataphylls or needles (Lee, 1952; Griffith, 1957; Kausik, 1975; Tomlinson *et al*., 1989; Dörken and Parsons, 2016; Dörken *et al*., 2021; Andruchow-Colombo *et al*., 2024). The species that we examined have no endodermis around their leaf veins or concentric vascular bundles in phylloclades or leaves.

#### Phyllocladus alpinus

A phylloclade (Dörken *et al*., 2021) is illustrated at mid-phylloclade level in Fig. 2J. Viewed in berberine (Fig. 2J) and berberine gentian violet (Fig. 2K), some of the vascular tissues are stem-like and arranged in a concentric fashion, hence concentric vascular bundles, with very few xylem elements and no endodermis (Fig. 2K); there may be one stem-like stele (concentric vascular bundles) or a few stem-like vascular bundles. Lateral veins generally have a few xylem elements without endodermis and are oriented perpendicular to the epidermis, not adaxially/abaxially (Fig. 2K). At the phylloclade base (Fig. 2L) there is a ring of vascular bundles, i.e. a stem stele, sometimes with sclerified cells and no endodermis. All veins and concentric vascular bundles are without vein sheath or endodermis (Dörken *et al*., 2021).

#### Dacrycarpus dacrydioides

For the needle-like ends of the cataphylls and their bases, there is no endodermis, but vein sheath cells occur (Fig. 2M). Dörken and Parsons (2016) presented no evidence of endodermis or vein sheath in their standard histochemical preparations.

#### Dacrydium elatum

In *D. elatum*, needle-like ends of the cataphylls (Fig. 2N) do not have an endodermis (Fig. 2O), but there are vein sheath cells in the cataphylls and their bases (Fig. 2N). In one of the few studies to look at petioles, Lee (1952, p. 396) similarly noted that there was no ‘endodermal sheath’ in the leaf or petiole. Dörken and Parsons (2016) also showed no evidence of endodermis or a vein sheath in *Dacrydium cupressinum*; however, there was some evidence of a vein sheath in the bright-field micrograph of a leaf in Sahni and Mitra (1927).

#### Podocarpus macrophyllus

Like Griffith (1957) or Kausik (1975), under berberine (Fig. 2P) and berberine gentian violet (Fig. 2Q) staining our specimens of *P. macrophyllus* had no endodermis or vein sheath in the needle or petiole (Fig. 2R). We did observe transfusion tissue under berberine but not under berberine gentian violet staining (Fig. 2P; see Griffith, 1957).

### Sciadopityaceae

#### Sciadopitys verticillata

The shoot structures on our plants were cladodes (Dörken and Stützel, 2011) in which two well-separated veins have an oblique orientation seen under berberine epifluorescence (Fig. 2S); there is no endodermis (Fig. 2T, berberine gentian violet) even at their base and no obvious vein sheath. Scale leaves have a very small vein with little to no xylem and no endodermis or vein sheath of lignified cells (Fig. 2U). The Dörken and Stützel (2011) bright-field images show that there is no evident ring of endodermis, but there is a vein sheath (or vein sheet, per Dörken and Stützel, 2011). The image of a fossil *Sciadopitys* (Stockey *et al*., 2005) shows two veins but no endodermal or sheath region.

### Cupressaceae

The leaves of members of the Cupressaceae have such a seeming variety of morphologies from phylloclades, needles and flattened laminae to cataphylls (Gifford and Foster, 1989; Kaplan, 2022) that we were not surprised to find that many researchers reported a variety of situations for their vascular tissues. For example, Cross (1940) showed no evidence of endodermis but did report a vein sheath in *Cryptomeria*, whereas Ivănescu *et al*. (2007) and Yousif and Shahbaz (2023) noted but did not demonstrate an endodermis in various species not covered herein; in addition, some of the vascular tissues photographed and referred to were stem vascular bundles, not leaf or phylloclade tissues. Fossil Cupressaceae with phylloclade-like morphologies have a wider central vein or probable concentric vascular bundles much like typical, extant phylloclade-bearing plants (Stockey *et al*., 2005; Dörken *et al*., 2023*a*, *b*; the current study for *Phyllocladus* of the Podocarpaceae and *Fokienia* of the Cupressaceae).

#### Cunninghamia lanceolata

**Fig. 3.**
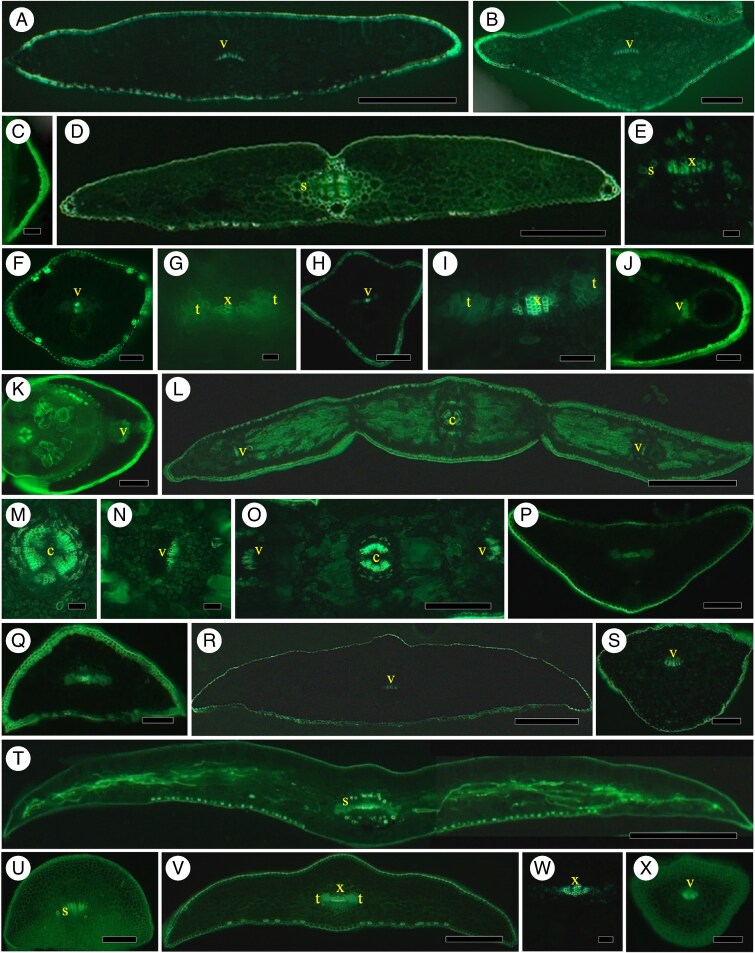
Cupressaceae and Taxaceae. (A, B) *Cunninghamia lanceolata*. (A) Needle, no endodermis or vein sheath, BGVef (see abbreviations list in legend of Fig. 1). Scale bar = 500 μm. (B) Petiole, no endodermis or vein sheath, Bef. Scale bar = 200 μm. (C) *Athrotaxis cupressoides*. Scale leaf, no endodermis or vein sheath. Scale bar = 125 μm. (D, E) *Metasequoia glyptostroboides*. (D) Needle transverse section, vein sheath, Bef. Scale bar = 400 μm. (E) Petiole vein with vein sheath, xylem. Scale bar = 100 μm. (F, G) *Sequoiadendron giganteum*. Needle transverse section, no obvious endodermis or vein sheath, BGVef. Scale bar = 215 μm. (G) Close-up of vein, xylem and transfusion tissue, BGVef. Scale bar = 70 μm. (H, I) *Cryptomeria japonica*. (H) Needle section, BGVef. Scale bar = 200 μm. (I) Vein, xylem, transfusion tissue, no endodermis or vein sheath, BGVef. Scale bar = 150 μm. (J) *Thuja occidentalis* cataphyll, no endodermis or vein sheath, BGVef. Scale bar = 150 μm. (K) *Chamaecyparis pisifera*. Vein of cataphyll against stem, Bef. Scale bar = 200 μm. (L–O) *Fokienia hodginsii*. (L) Mid-phylloclade section, concentric vascular bundles at c, two lateral veins at v, BGVef. Scale bar = 500 μm. (M) Concentrix vascular bundles in centre of phylloclade, no endodermis. Scale bar = 100 μm. (N) One of two veins of phylloclade, no endodermis, BGVef. Scale bar = 60 μm. (O) Phylloclade base, two veins nearer reorientated concentric vascular bundles, no endodermis, BGVef. Scale bar = 300 μm. (P) *Juniperus chinensis* cataphyll, no endodermis or vein sheath, BGVef. Scale bar = 200 μm. (Q) *Juniperus horizontalis* cataphyll, no endodermis or vein sheath, xylem and transfusion tissue, Bef. Scale bar = 200 μm. (R, S) *Cephalotaxus harringtonia*. (R) Needle, no endodermis or vein sheath around vein, BGVef. Scale bar = 450 μm. (S) Petiole, vein without endodermis or vein sheath, BGVef. Scale bar = 150 μm. (T, U) *Amentotaxus formosana*. (T) Broad leaf with one vein, xylem, transfusion tissue and partial vein sheath, Bef. Scale bar = 700 μm. (U) Petiole, vein with partial vein sheath, BGVef. Scale bar = 300 μm. (V–X) *Taxus cuspidata*. (V) Needle, xylem, transfusion tissue, no apparent endodermis or sheath, Bef. Scale bar = 400 μm. (W) Close-up of xylem, no endodermis or vein sheath, BGVef. Scale bar = 75 μm. (X) Petiole, vein, no endodermis or vein sheath, Bef. Scale bar = 150 μm.

Neither needles (Fig. 3A) nor petioles (Fig. 3B) have endodermis or a distinctive, lignified vein sheath in *C. lanceolata*. Cross (1940) showed no endodermis but noted a vein sheath without good evidence (even referring to a wrong photograph).

#### Athrotaxis cupressoides

The tightly positioned, scale-like cataphylls of *A. cupressoides* lack an endodermis and vein sheath (Fig. 3C). Only a faint vein sheath was reported by Napp-Zinn (1966, noting a 19th-entury report).

#### Metasequoia glyptostroboides

The needle (Fig. 3D) and petiolar end of the needle (Fig. 3E) in *M. glyptostroboides* have no endodermis, but they do have a vein sheath in needles and petiolar regions. Sterling (1949, p. 468) stated that ‘No distinct endodermal layer is present’. Standard preparations and micrographs in Ng and Smith (2020) reveal a possible vein sheath, but no endodermis, including in fossil *Metasequoia*. In *Metasequoia* roots, however, endodermis and exodermis have been clearly demonstrated (Yang *et al*., 2019).

#### Sequoiadendron giganteum

We found no endodermis or vein sheath in the needle-like end of the cataphylls (Fig. 3F, G, with transfusion tissue) or at the bases of cataphylls of *S. giganteum*. Chin and Sillett (2016) showed no endodermis, but they did demonstrate fibres around the vein of a cataphyll.

#### Sequoia sempervirens

There is no endodermis or vein sheath in needles (Fig. 1G) or petioles (Fig. 1H) of *S. sempervirens*. After Napp-Zinn (1966, noting far earlier researchers) noted only faint vein sheath in *Sequoia*, photographs in Oldham *et al*. (2010) and Chin *et al*. (2022) did not reveal any evidence of endodermis, although a vein sheath or fibres may have been present.

#### Cryptomeria japonica

Our sections of *C. japonica* did not reveal an endodermis or vein sheath with lignified cell walls in either the needle (Fig. 3H) or the petiolar base, both with xylem and transfusion tissues (3I). Cross (1940, p. 577) stated that an ‘incipient endodermal sheath may be present’. Azuma *et al*. (2015) labelled a vein sheath in needle leaves of *C. japonica*, but they did not reveal sclerification.

#### Thuja occidentalis

Where cataphylls arise from stems and partially surround the stem (Fig. 3J), they have no endodermis or vein sheath. Dörken (2013*a*) also did not find endodermis or vein sheath in *Thuja plicata*.

#### Chamaecyparis pisifera

*Chamaecyparis* has cataphylls that terminate in a needle-like tip; neither their needle-like portions (Fig. 3K) nor bases have an endodermis or vein sheath, although there is transfusion tissue in the vein (Shiraki *et al*., 2016). Ivănescu *et al*. (2007) noted, without demonstrating, that its leaves had endodermis.

#### Fokienia hodginsii

The photosynthetic organs of *F. hodginsii* are phylloclades (Fig. 3L). The central vascular tissue comprises stem-like concentric vascular bundles (Fig. 3L, M; also see Dörken *et al*., 2021), and the two lateral vascular strands are leaf veins (Fig. 3L, N), each with a single bundle of xylem; these are arranged with xylem lateral to phloem rather than in an adaxial/abaxial pattern without endodermis or vein sheath (Fig. 3N). Near phylloclade bases, the veins have their perpendicular-to-epidermis orientation, but the stem vasculature is opposite that found in the phylloclade (Fig. 3O); phylloclades lack endodermal Casparian bands or vein sheaths (Fig. 3L–O). The phylloclade pattern was shown long ago by Saxton (1930) for *Fokienia*, with a variation of the position of the two lateral veins in *Libocedrus*. While *Fokienia* has been found to be very closely related to *Chamaecyparis* and *Thuja* of the Cupressaceae (Lu *et al*., 2014; Zang *et al*., 2019), its leaves are clearly more similar structurally to *Phyllocladus* of the Podocarpaceae (Zang *et al*., 2019; Dörken *et al*., 2021); phylloclades were termed branchlets by Saxton (1930), and Dörken *et al*. (2021) clarified their nature and structures. These phylloclades in *Fokienia* are not like the leaves or cataphylls of *Chamaecyparis* or *Thuja*. Yousif and Shahbaz (2023) had bright-field micrographs of cupressaceous plants with stem-like vasculature in the leaves of *Platycladus*; they also stated that an endodermis was present in these genera, including around secretory canals, but an endodermis was not revealed in their micrographs.

#### Juniperus

We did not find an endodermis or vein sheath in the needle-like or scale leaves and petiolar regions of *J. chinensis* (Fig. 3P) or *J. horizontalis* (Fig. 3Q). Güvenc *et al*. (2011) labelled endodermis in standard preparations of *Juniperus* species, but there was no evidence of Casparian bands.

### Cephalotaxaceae

#### Cephalotaxus harringtonia

Neither the needle (Fig. 3R) nor the petiole (Fig. 3S) has an endodermis or vein sheath in *C. harringtonia*. Similarly, Biswas and Johri (1997) stated that the lone vein does not have a vein sheath in *C. fortunei*, nor did Hu (1984) reveal endodermis in *Cephalotaxus*, although transfusion tissue was present. However, the autofluorescence photograph in Elpe *et al*. (2017) has an apparent vein sheath.

### Taxaceae

Ghimire *et al*. (2014, p. 379) found an endodermis ‘scarcely evident’ or ‘parenchymatous’ in the Taxaceae, yet they labelled an endodermis in *Taxus*. Photographs in Elpe *et al*. (2017) reveal a possible vein sheath in *Taxus*, *Pseudotaxus* and *Torreya*.

#### Amentotaxus formosana

The leaf lamina has no endodermis, but there are vein sheath cells in the leaf lamina (Fig. 3T) and petiole (Fig. 3U); the extension of sheath fibres/sclerenchyma throughout the lamina from the single vein ending is striking (Fig. 3T). For *A. formosana*, Chin and Lin (2022, p. 4) also identified a vein sheath around the singular vein, as well as a vascular cambium in the vein and the extension of transfusion cells with ‘elongated sclerenchyma’ throughout the mesophyll (see also Ghimire *et al*., 2014). Elpe *et al*. (2017) labelled some cells as vascular sclereids in *Amentotaxus argotaenia* and *Torreya californica*, but did not demonstrate endodermis or vein sheath.

#### Taxus cuspidata

In *Taxus*, the needle (Fig. 3V, W) and petiole (Fig. 3X) have no endodermis or vein sheath (Roden *et al*., 2015), but transfusion tissue is readily apparent (Fig. 3V). Gostin and Predan (2022) found no endodermis in bright-field images, but Ghimire *et al*. (2014) labelled an endodermis in bright-field sections, but no Casparian bands were visible. Elpe *et al*. (2017) showed a possible vein sheath in *T. baccata* and *Pseudotaxus chienii* in fluorescence microscopy.

### Pinaceae

Dörken and Stützel (2012) and Dörken *et al*. (2010) have demonstrated with standard preparation techniques and bright-field microscopy an endodermis in many members of the pines. Interestingly, Du *et al*. (2020, p. 1) categorized the differences between the ‘flattened leaves’ of *Abies*, *Larix*, *Pseudolarix* and *Pseudotsuga* and the ‘needlelike leaves’ of *Cedrus*, *Picea* and *Pinus* in regard to photosynthetic mesophyll capacity and adaxial/abaxial tissue polarity, but endodermis presence does not fully follow their categories; our findings below will show that only certain species of *Pinus* have oblique vein orientations. A bright-field image of *Cathaya* leaf shows an apparent endodermis (Hu and Wang, 1984); Pant and Basu (1977) show Casparian bands only in drawings. Fossilized needles of *Pinus foisyi* (Miller, 1992) demonstrate endodermis.

#### Cedrus atlantica

**Fig. 4.**
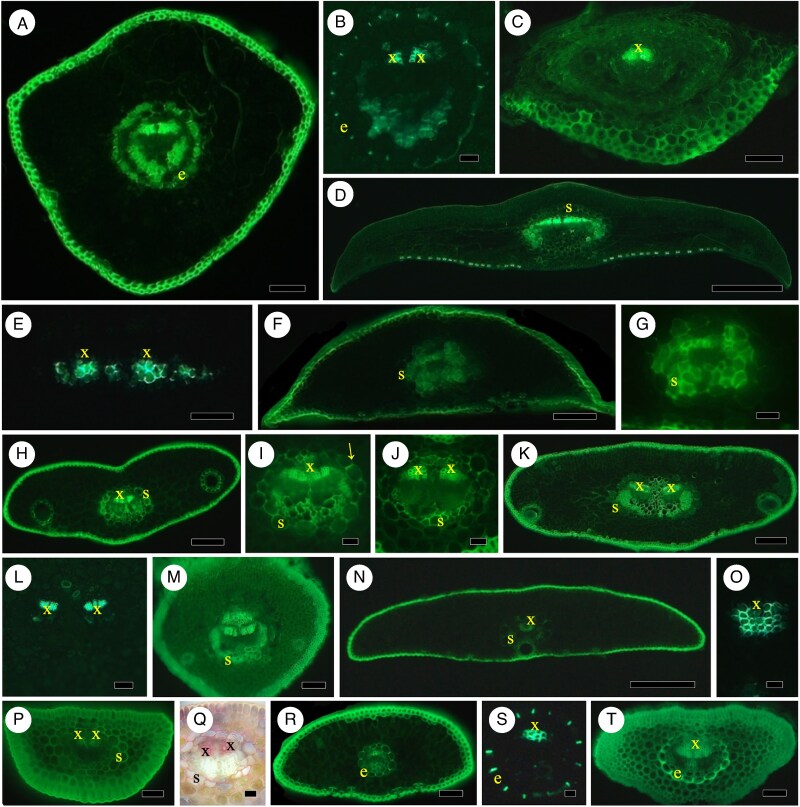
Pinaceae. (A–C) *Cedrus atlantica*. (A) Needle, endodermis vein, Bef (see abbreviations list in legend of Fig. 1). Scale bar = 400 μm. (B) Vein with Casparian bands in endodermis, BGVef. Scale bar = 40 μm. (C) Petiole, no endodermis, Bef. Scale bar = 125 μm. (D–F) *Keteleeria davidiana.* (D) Needle transverse section, vein, no endodermis, vein sheath, Bef. Scale bar = 400 μm. (E) Needle vein, xylem, no endodermis, BGVef. Scale bar = 100 μm. (F) Petiole vein with vein sheath, Bef. Scale bar = 200 μm. (G) Close-up of vein, vein sheath, Bef. Scale bar = 60 μm. (H–J) *Abies balsamea*. (H) Section of needle, no endodermis, vein sheath, Bef. Scale bar = 200 μm. (I) Close-up of vein, vein sheath with arrow on radial cell wall, Bef. Scale bar = 60 μm. (J) Close-up of petiole vein, vein sheath, xylem, no endodermis, Bef. Scale bar = 60 μm. (K–M) *Abies concolor*. (K) Two-veined needle, vein sheath, Bef. Scale bar = 225 μm. (L) Close-up of two veins, no endodermis, some vein sheath cells visible. Scale bar = 75 μm. (M) Petiole, two veins with vein sheath, Bef. Scale bar = 125 μm. (N–Q) *Tsuga canadensis*. (N) Needle, vein sheath around vein, unstef. Scale bar = 350 μm. (O) Close-up of vein xylem without endodermis, BGVef. Scale bar = 60 μm. (P) Petiole, two veins, no endodermis, vein sheath, unstef. Scale bar = 100 μm. (Q) Petiole, two veins, lignified cells of vein sheath, Pgbf. Scale bar = 60 μm. (R–T) *Pseudotsuga menziesii*. (R) Needle, vein surrounded by endodermis, Bef. Scale bar = 200 μm. (S) Close-up of vein with endodermis Casparian bands, BGVef. Scale bar = 30 μm. (T) Petiole, xylem of vein, endodermis with Casparian bands and thickened outer tangential walls, Bef. Scale bar = 100 μm.

*Cedrus atlantica* has a clearly demonstrable endodermis with Casparian bands in its needles (Fig. 4A, B), but in petioles there are no Casparian bands or vein sheaths (Fig. 4C); veins are oriented adaxially/abaxially. In bright-field images of needles, Fahn (1990) clearly demonstrated endodermal Casparian bands in *C. deodara*, and Bercu *et al*. (2010) and Bakkali and Amraoul (2022) illustrated endodermis with Casparian bands in *C. atlantica*.

#### Keteleeria davidiana

In our specimens of *K. davidiana*, there is a vein sheath in needles (Fig. 4D) and petioles (Fig. 4F), but no demonstrable endodermis with Casparian bands in needles (Fig. 4E) or petioles (Fig. 4F, G). Pant and Basu (1977) showed only drawings of the ring of endodermis in *Keteleeria*. Pant and Basu (1977) noted that there was an endodermis with Casparian bands, suberin and lignified secondary walls, but these were not apparent in their photographs of the closely related *Cathaya*, only in drawings with a ring of endodermis. Hu and Wang (1984) noted Casparian bands without demonstrating them in *Cathaya* needles, but not in petioles; a ring of cells in the position of endodermis was visible in both leaf parts. Han (1984) showed only an incomplete ring of endodermis without identifiable Casparian bands in *Keteleeria*, but no such ring of endodermis was in *Cathaya*.

#### Abies

Around the two veins of needles, *A. balsamea* has a vein sheath with some lignified cell walls (Fig. 4H) even on radial walls in the position of Casparian bands (Fig. 4I, arrow), but no distinct endodermis. In petioles, there is a vein sheath without endodermis (Fig. 4J). *Abies concolor* has a vein sheath around the two veined needles without endodermis (Fig. 4K) and in petioles (Fig. 4M); Casparian bands via berberine gentian violet epifluorescence are not revealed in either needles (Fig. 4L) or petioles. Dörken (2014) and Dörken and Lepetit (2018) illustrated a vein sheath in the related *A. alba*, although others (e.g. Bercu *et al*., 2010; Ghimire *et al*., 2015*a*) illustrated endodermis, but Casparian bands were not evident. Endoh *et al*. (2012) had a ring of endodermis under bright-field, but Casparian bands were not visible in cryo-SEM images.

*Tsuga canadensis* needles (Fig. 4N) and petioles (Fig. 4P, Q) have a vein sheath without endodermis (Fig. 4O). There is usually just one vein in needles, although it may appear somewhat divided, whereas petioles have greater division between two units of the xylem. Soar (1922) showed lignified and suberized endodermal cells in a drawing, and Gambles and Dengler (1974) clearly demonstrated a partial vein sheath in *T. canadensis*.

#### Pseudotsuga menziesii

Needles (Fig. 4R, S) and petioles (Fig. 4T) have distinct endodermis Casparian bands (Fig. 4S); they also have lignified outer tangential secondary walls without suberin lamellae (Fig. 4T). Soar (1922), Pădure *et al*. (2008), Chin and Sillett (2019) and Apple *et al*. (2002) also demonstrated the Casparian bands in this species.

#### Larix

**Fig. 5.**
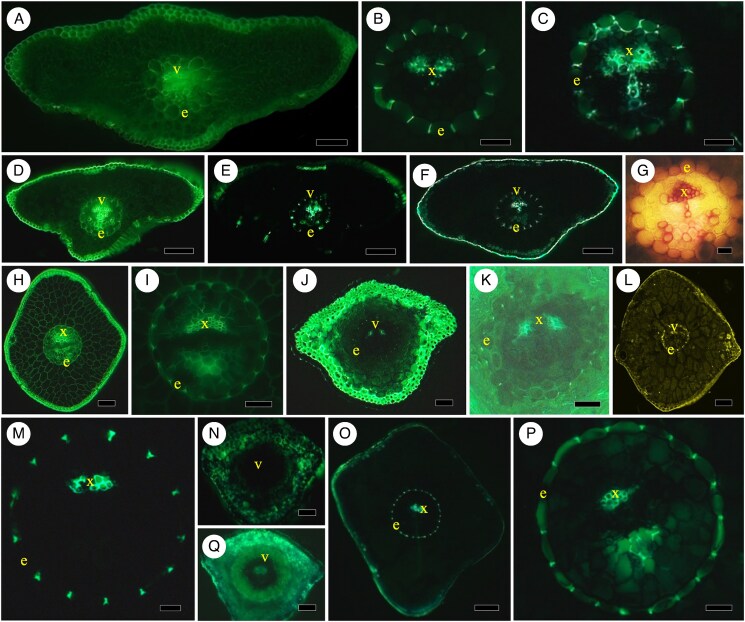
Pinaceae. (A–C) *Larix laricina*. (A) Needle, endodermis around vein, Bef (see abbreviations list in legend of Fig. 1). Scale bar = 100 μm. (B) Close-up of vein with endodermis Casparian bands, BGVef. Scale bar = 60 μm. (C) Close-up of needle base, vein with Casparian bands of endodermis, BGVef. Scale bar = 50 μm. (D, E) *Larix kaempferi*. (D) Needle, endodermis around vein, Bef. Scale bar = 150 μm. (E) Base of needle with endodermis Casparian bands, BGVef. Scale bar = 175 μm. (F, G) *Larix occidentalis*. (F) Needle, endodermis with Casparian bands around vein, BGVef. Scale bar = 130 μm. (G) Needle base, endodermis with Casparian bands and outer tangential walls of endodermal cells stained for lignin, Pgbf. Scale bar = 50 μm. (H–K) *Picea abies*. (H) Needle, one vein, endodermis, Bef. Scale bar = 150 μm. (I) Needle vein, endodermis Casparian bands. Scale bar = 75 μm. (J) Distal petiole with endodermis, Bef. Scale bar = 120 μm. (K) Distal petiole to show Casparian bands of endodermis, Bef. Scale bar = 70 μm. (L–N) *Picea glauca*. (L) Needle, endodermis around vein, BGVlc. Scale bar = 150 μm. (M) Needle vein, endodermis Casparian bands, BGVef. Scale bar = 35 μm. (N) Petiole, no endodermis, BGVef. Scale bar = 1000 μm. (O–Q) *Picea pungens*. (O) Needle, endodermis, BGVef. Scale bar = 220 μm. (P) Needle vein, endodermis Casparian bands, BGVef. Scale bar = 45 μm. (Q) Petiole, no endodermis, Bef. Scale bar = 100 μm.

In *L. laricina*, endodermis with Casparian bands is present in needles (Fig. 5A, B) and leaf bases or petioles (Fig. 5C) with some evidence of lignified tangential walls in needles in berberine staining (Fig. 5A, low magnification). In *L. kaempferi* (Fig. 5D, E) and *L. occidentalis* (Fig. 5F, G) endodermis is present in needles and leaf bases (petioles); in *L. kaempferi* outer tangential endodermal cell walls were thickened under low magnification in berberine staining (Fig. 5D) and in *L. occidentalis* at higher magnification under phloroglucinol staining (Fig. 5G). Endodermis in *Larix* has been shown by Eguchi *et al*. (2004) and Bercu and Popoviciu (2013) without demonstrable Casparian bands.

*Picea abies*, *P. glauca* and *P. pungens* In the three species of *Picea* studied here, *P. abies* has endodermis in the needles (Fig. 5H, I) and distal petioles (Fig. 5J, K), but not in proximal petioles; the outer tangential walls fluoresce lightly in berberine (Fig. 5H). *Picea glauca* has distinctive endodermis in needles (Fig. 5L, M), but no endodermis or vein sheath in petioles (Fig. 5N), and *P. pungens* also has endodermis with Casparian bands in needles (Fig. 5O, P), but no endodermis or vein sheath in petioles (Fig. 5Q). Endodermis was demonstrated in *P. abies* by Soukupová *et al*. (2000), in *P. glauca* by Hacke *et al*. (2015) and in *P. pungens* by Soukupová *et al*. (2001) and Qin *et al*. (2014). Endodermis was also shown in needles of *Picea* species by Ghimire *et al*. (2015*a*) without evident Casparian bands.

#### Pinus

**Fig. 6:**
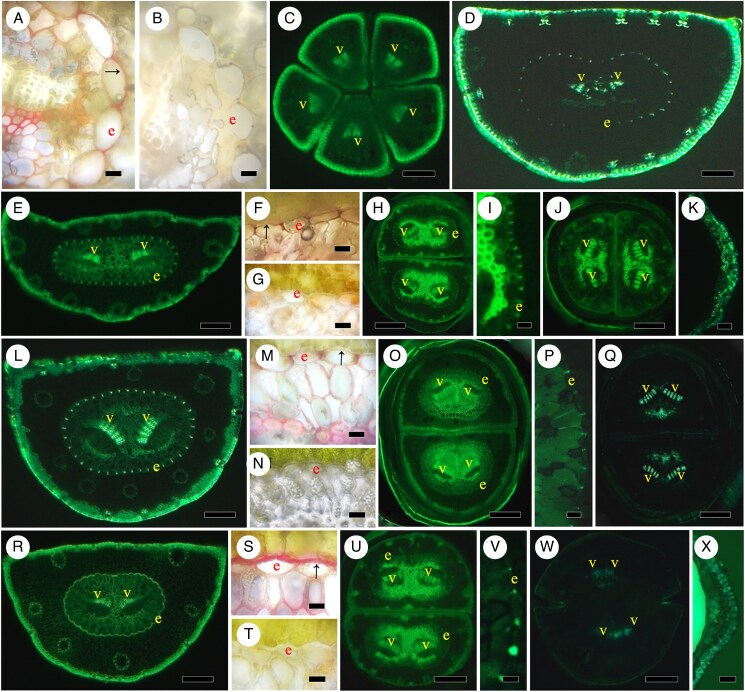
(A–C) *Pinus. strobus* (see also Fig. 1A–C). (A) Needle, part of vein, endodermis with slightly lignified outer walls (arrow), Pgbf (see abbreviations list in legend of Fig. 1). Scale bar = 30 μm. (B) Part of vein, endodermis with no evidence of suberin lamellae, SR7Bbf. Scale bar = 35 μm. (C) Fascicle five-needle bases, no endodermis Casparian bands, Bef. Scale bar = 300 μm. (D) *Pinus mugo.* Needle, endodermis Casparian bands, Bef. Scale bar = 150 μm. (E–K) *Pinus sylvestris*. (E) Needle, two distinct veins, endodermis ring Casparian bands, Bef, Scale bar = 350 μm. (F) Needle, part of vein, endodermis with slightly lignified outer tangential walls (arrow), Pgbf. Scale bar = 35 μm. (G) Part of vein, endodermis with no evidence of suberin lamellae, SR7Bbf. Scale bar = 30 μm. (H) Distal fascicle of two needles, endodermis, Bef. Scale bar = 150 μm. (I) Close-up of part of endodermis with Casparian bands from Fig. 5V, BGVef. Scale bar = 50 μm. (J) Proximal fascicle needles with two veins each, no endodermis. Scale bar = 150 μm. (K) Scale leaf, vein with no endodermis and no visible xylem. Scale bar = 70 μm. (L–Q) *Pinus nigra*. (L) Transverse section of needle with two veins, endodermis with distinct Casparian bands, Bef. Scale bar = 150 μm. (M) Needle, part of vein, endodermis with Casparian bands with slightly thickened outer walls, Pgbf. Scale bar = 35 μm. (N) Part of vein, endodermis with no evidence of suberin lamellae, SR7Bbf. Scale bar = 30 μm. (O) Distal fascicle of two needles, endodermis, Bef. Scale bar = 150 μm. (P) Close-up from Fig. 5O to show Casparian bands in distal fascicle needle, BGVef. Scale bar = 50 μm. (Q) Proximal fascicle with no endodermis, BGVef. Scale bar = 150 μm. (R–Y) *Pinus resinosa*. (R) Needle transverse section, endodermis with thickened outer tangential cell walls, Bef. Scale bar = 150 μm. (S) Needle, part of vein, endodermis with slightly lignified outer walls, Pgbf. Scale bar = 35 μm. (T) Part of vein, endodermis with no evidence of suberin lamellae, SR7Bbf. Scale bar = 30 μm. (U) Distal fascicle of two needles, endodermis, Bef. Scale bar = 150 μm. (V) Close-up of part of endodermis from Fig. 5V, BGVef. Scale bar = 35 μm. (W) Proximal fascicle needles with two veins each, no endodermis. Scale bar = 150 μm. (X) Scale leaf, no endodermis, vein with no visible xylem. Scale bar = 70 μm.

*Pinus strobus* (Fig. 1A–C) has single vein needles with a distinctive endodermis with Casparian bands (Figs 1B, C and 6A) and some lignification on tangential cell walls (Fig. 6A) but no suberin lamellae (Fig. 6B); the xylem of the vein is often in two slightly separated units (Fig. 1C). In the needle bases within the middle-lower fascicle or petiolar region, there is no endodermis or vein sheath (Fig. 6C); there is endodermis in the most distal petiolar regions (not shown). *Pinus mugo* needles have an endodermis with Casparian bands around the two veins (Fig. 6D); the fascicle base has two petiolar needles without endodermis or vein sheath around the veins (not shown). In *P. sylvestris*, the two-veined needles have a distinctive endodermis with lignified Casparian bands (Fig. 6E, F) but no suberin lamellae (Fig. 6G); the outer tangent wall is thickened but stained unevenly for lignin (Fig. 6F). Casparian bands are found in distal fascicled needles (Fig. 6H, I) but not in proximal fascicled needles (Fig. 6J), indicating that endodermis has developed before needles become separate in fascicles. Scale leaves have no endodermis and often show no xylem (Fig. 6K).

In *P. nigra* leaves, needles have endodermis with Casparian bands around the two veins (Fig. 6L); Casparian bands stain for lignin (Fig. 6M, on either side of arrow or e), but outer tangential cell walls do not stain for lignin via phloroglucinol HCl in bright-field or berberine gentian violet in epifluorescence, although they appear thicker (Fig. 6M at arrow). There is no evidence of suberin lamellae (Fig. 6N). In unstained specimens viewed in epifluorescence (Fig. 6O, difficult to see) distal and partially separated needles within a fascicle develop an endodermis with Casparian bands (Fig. 6P). In proximal, unseparated needles within the fascicle there is no endodermis in the two needles (Fig. 6Q). *Pinus resinosa* has mature needles with two veins surrounded by an endodermis (Fig. 6R) with Casparian bands (Fig. 6R, S) and distinctly secondarily thickened outer tangential cell walls of lignin (Fig. 6R, S); they do not have suberin lamellae (Fig. 6T). Distal, slightly separated needles within a fascicle have Casparian bands in endodermis around each two-veined needle (Fig. 6U, V), whereas proximal or petiolar needles in fascicles do not have endodermis (Fig. 6W). Scale leaves have little to no xylem and no endodermis (Fig. 6X). In three species of *Pinus* with two veins within an endodermis we find varying obliquely oriented veins (Fig. 6E, L, R; cf. Du *et al*., 2020).

Chamberlain (1935), Keng and Little (1961), the Dörken group (e.g. Dörken and Stützel, 2012) and Kaplan (2022), have had illustrations of pine needles with an endodermis. Kuusk (2024) has recently shown bright-field images of needles of *P. nigra* that seem to have more lignin in their endodermal cell walls after 2 years than in their first-year needles after phloroglucinol HCl treatment. Endodermis in *P. sylvestris* showed clearly in images via laser confocal images in Lin *et al*. (2001) and in bright-field by Dörken and Stützel (2012). No cataphyll endodermis was revealed in bright-field images in *P. lambertiana* by Sacher (1955). Carde (1978) questioned the occurrence of Casparian bands in *P. pinaster* needles, but Gao *et al*. (2023) clearly demonstrated Casparian bands. Like Meicenheimer *et al*. (2008) for *P. resinosa*, Donaldson and Williams (2018) demonstrated unequivocally that there were secondarily lignified outer tangential cell walls in *P. radiata*, but their fluorescence photographs also showed clearly Casparian bands without suberin lamellae in the endodermis using techniques like ours. The bright-field images of various *Abies*, *Picea* (Ghimire *et al*., 2015*a*) and *Pinus* species (e.g. *P. sylvestris*, *P. parviflora*, *P. densiflora*, *P. taeda*; Ghimire *et al*., 2015*b*) also showed thickened outer tangential endodermal cell walls. Miller (1992) also demonstrated clearly thickened outer tangential cell walls in the fossilized endodermis of *P. foisyi*. In *P. nigra*, Meicenheimer *et al*. (2008) had bright-field and fluorescent sections of *P. nigra* with no secondary cell walls, but Lux *et al*. (2017) and many others (e.g. Ghimire *et al*., 2015*a*, 2015*b*; Donaldson and Williams, 2018 (*P. radiata*)) had bright-field photographs with distinct outer tangential cell walls that were thickened and lignified. Soar (1922) illustrated (but did not present photographs of) the lack of endodermis in the proximal regions of the fascicled base of pines and its presence developing prior to emergence of the needles between the scale leaves of fascicles. Few other researchers have determined the presence or absence of endodermis or vein sheaths in petioles or scale leaves in pines.

## DISCUSSION

As shown in Table 1, an endodermis with Casparian bands is found around leaf veins only in the Pinophyta family Pinaceae, where it is found in most members (*Cedrus atlantica*; *Pseudotsuga menziesii*; *Larix laricina*, *L. kaempferi* and *L. occidentalis*; *Picea abies*, *P. glauca and P. pungens*; *Pinus mugo*, *P. nigra* and *P. resinosa* with lignified secondary outer tangential walls, *P. strobus* and *P. sylvestris*). Petioles, in contrast, almost always lack endodermis; the notable exceptions with Casparian bands in petiolar regions are *Pseudotsuga menziesii*, *Larix laricina*, *Picea abies*, and distal petiolar regions within fascicles of *Pinus nigra*, *P. resinosa*, *P. strobus* and *P. sylvestris*. Within the 11 genera of Pinaceae and the 8 we studied, only needles of *Keteleeria*, *Abies* and *Tsuga* lack distinctive Casparian bands in an endodermis-like ring around veins, and only these same three Pinaceae genera have vein sheaths. Both *Keteleeria* and *Abies* are interesting because their needles and petioles showed indications of cell wall modifications that might represent incipient endodermis. Thus, in detail, with our study and with other examples in the literature, we have resolved the issues of Napp-Zinn (1966) and Lersten (1997) that endodermis with Casparian bands around veins had not been positively identified in needles of many members of the Pinaceae.

**Table 1.** The presence of endodermis and vein sheath in representative gymnosperms.

Phylum and family	Species	Endodermis with Casparian bands		Vein sheath	
		Leaf	Petiole	Leaf	Petiole
Cycadophyta, Cycadaceae	*Cycas revoluta*			✓	✓
Ginkgophyta, Ginkgoaceae	*Ginkgo biloba*			✓	✓
Pinophyta, Araucariaceae	*Araucaria araucana*				
Pinophyta, Podocarpaceae	*Phyllocladus alpinus*				
	*Dacrycarpus dacrydioides*			*	*
	*Dacrydium elatum*			*	*
	*Podocarpus macrophyllus*				
Pinophyta, Sciadopityaceae	*Sciadopitys verticillata*				
Pinophyta, Cupressaceae	*Cunninghamia lanceolata*				
	*Athrotaxis cupressoides*				
	*Metasequoia glyptostroboides*			✓	✓
	*Sequoia sempervirens*				
	*Sequoiadendron giganteum*				
	*Cryptomeria japonica*				
	*Thuja occidentalis*				
	*Chamaecyparis pisifera*				
	*Fokienia hodginsii*				
	*Juniperus horizontalis*				
	*Juniperus chinensis*				
Pinophyta, Cephalotaxaceae	*Cephalotaxus harringtonia*				
Pinophyta, Taxaceae	*Amentotaxus formosana*			*	*
	*Taxus cuspidata*				
Pinophyta, Pinaceae	*Cedrus atlantica*	✓			
	*Keteleeria davidiana*			✓	✓
	*Abies balsamea*			✓	✓
	*Abies concolor*			✓	✓
	*Tsuga canadensis*			✓	✓
	*Pseudotsuga menziesii*	✓	✓		
	*Larix occidentalis*	✓	✓		
	*Larix kaempferi*	✓	✓		
	*Larix laricina*	✓	✓		
	*Picea abies*	✓	✓		
	*Picea pungens*	✓			
	*Picea glauca*	✓			
	*Pinus nigra*	✓	✓		
	*Pinus resinosa*	✓	✓		
	*Pinus strobus*	✓	✓		
	*Pinus sylvestris*	✓	✓		
	*Pinus mugo*	✓			
Gnetophyta, Gnetaceae	*Gnetum gnemon*				
Gnetophyta, Ephedraceae	*Ephedra tweedieana*				

Taxa are listed according to Yang *et al*. (2022).

^*^Partial vein sheath.

In contrast to endodermis, vein sheaths are prominent in the most early evolved gymnosperms, namely Cycadophyta and Ginkgophyta, but they are present in relatively few members of the Pinophyta and are absent in the Gnetophyta. Viewed in current phylogenies (Gernandt *et al*., 2016; Yang *et al*., 2022), the distribution of this trait (Table 1) suggests a loss in several lineages or several independent origins. A few gymnosperms in the Pinophyta families of Podocarpaceae (*Dacrycarpus dacrydioides* and *Dacrydium elatum*), Cupressaceae (*Metasequoia glyptostroboides*) and Taxaceae (*Amentotaxus formosana*) have vein sheaths or sclerified cells in positions of possible vein sheaths. The derived lineage Gnetophyta (*Gnetum gnemon* and *Ephedra tweedieana*) has no endodermis or vein sheath in leaves.

If we consider the fossil record (Taylor *et al*., 2009), for example, Robison (1977) had micrographs of fossil *Pinus* fascicles to show that an endodermis, without visible Casparian bands, was present in distal needles bases, but not proximal bases. He also claimed that there was only a thick-walled sheath in an earlier fossil, *Prepinus* (Robison, 1974). Then, Stockey and Ueda (1986) produced light micrographs of five-needled fossil *Pinus hokkaidoensis* that clearly reveal a ring of endodermal cells without visible Casparian bands around the vein. Taylor *et al*. (2009, in a photograph by R. A. Stockey) had a fossil five-needle pine, *P. hokkaidoensis,* like *P. strobus*, with a quite distinct endodermis ring around the vein in each needle with distinctly thickened radial and outer tangential walls. The absence of an endodermis with Casparian bands in the Abietoideae of the Pinaceae fossil records supports the idea that it is a more recently evolved structure and that it may have evolved multiple times. It would help to have more anatomical images of fossil members of the Abietoideae and Pinoideae of the Pinaceae to track the evolution of endodermis with Casparian bands. The fossil record of other families like the Cupressaceae seems particularly difficult for obtaining a clear picture of internal structure with vein sheaths or no vein-encircling structures (e.g. Taylor *et al*., 2009; Herrera *et al*., 2017), complicating the task of tracking the evolution of vein sheath across gymnosperms.

Soar (1922) was among the first to discuss any physiological and ecological roles of Pinaceae leaves in enabling them to withstand temperatures in alpine and subalpine regions. She described the leaves as xerophytic in nature, with defined endodermis, placing them in contrast with angiosperm leaves where there is no endodermis. The development and structure of endodermis with Casparian bands in needles/leaves of the members of Pinaceae could have contributed to their ability to evolve in, colonize and radiate into extreme environments, especially in cold, high-elevation environments where other gymnosperms are not found. Indeed, members of Pinaceae such as *Picea* and *Pinus* are the typical treeline species in many mountain ranges around the world (e.g. Kaku, 1971; Skärby *et al*., 1998; Miller, 1999; Wu *et al*., 2005; Taylor *et al*., 2009; Roden *et al*., 2009, 2015; Leslie *et al*., 2018; Stegner *et al*., 2023) where blowing ice and rime ice, consequent desiccation and extreme low temperatures limit other species (e.g. Kaku, 1971; Skärby *et al*., 1998; Wu *et al*., 2005; Roden *et al*., 2009, 2015; Taylor *et al*., 2009; Stegner *et al*., 2023). As Wu *et al*. (2005) have importantly shown, endodermis with Casparian bands in needles of *Pinus bungeana* is more permeable than endodermis with Casparian bands in roots; a salient reason could be the lack of suberin lamellae as we have shown for other species of *Pinus*. In *Picea mariana*, Way and Sage (2008) showed temperatures had a pronounced effect upon vein and leaf size, but it was not apparent in their photographs if there were any effects upon the obvious endodermis which we have shown in other *Picea* species. Endoh *et al*. (2012) showed in a species of *Abies* that changes in needles were structurally visible during freezing events. Freeze tolerance gained through an endodermis with Casparian bands is a logical driver of its evolution and persistence in Pinaceae.

The lack of a clear, distinct endodermis in *Keteleeria* and *Abies* of Abietoideae could relate to their subtropical habitat and origin (Zhang *et al*., 2023) – and a possible lack of selection pressure for endodermis, potentially explaining why this anatomical feature is absent in these lineages but present in *Cedrus* of Abietoideae and members of Pinoideae. The possible incipient endodermis seen in these genera could suggest an intermediate evolutionary step between vein sheaths and endodermis. Selection pressure for the development of endodermis is understandable for species like *Abies balsamea*, which is a treeline species subject to extreme cold temperatures.

This relationship between leaf structures and the plant environments of the gymnosperm has also been studied with regard to levels of CO_2_ (Sage and Khoshravesh, 2016), as well as nutrients and other compounds. In terms of the control and facilitation of movements of nutrients, phosphates and gases through needle veins, endodermis and mesophyll, Gao *et al*. (2023) have demonstrated the importance of endodermis with Casparian bands (see also Uchida *et al*., 2012). Other researchers have shown how photosynthetic and respiratory processes with CO_2_ and O_2_ movements could be salient in considering how the endodermis functions in conifer leaves (e.g. Sutinen *et al*., 2000; Eguchi *et al*., 2004; Roden *et al*., 2015; Sage and Khoshravesh, 2016). These interactions and relationships between leaf structural and environmental effects need to be examined far more thoroughly. Indeed, this goes beyond normal leaf functions to the effects of air pollution on these gymnosperm leaves in which there is endodermis – very evident in bright-field images of *Pinus nigra* by Delian and Săvulescu (2022).

As noted in the Results, members of the Pinaceae generally have a State I endodermis with Casparian bands only, i.e. there are no suberin lamellae in the endodermis cells, as per Seago *et al*. (1999), Enstone *et al*. (2003), Soukup and Tylová (2018) and Yang *et al*. (2019). The occurrence of a secondarily lignified outer tangential wall might seem to make these cells more like an exodermis, except that they are lacking suberin lamellae. However, a typical exodermis has Casparian bands and suberin produced concomitantly in plant roots (Pazourek and Votrubová, 1997; Seago *et al*., 1999; Seago, 2002; Enstone *et al*., 2003; Schreiber and Franke, 2011; Bonacorsi and Seago, 2016; Soukup and Tylová, 2018) or in plant stems (Seago and Fernando, 2013; Bonacorsi and Seago, 2016). Where produced, secondary cell wall lignification between suberin lamellae and protoplasm in these cells is normally produced in the innermost tangential cell wall of endodermis and the outermost cell wall in exodermis (e.g. Meyer *et al*., 2009), as found in gymnosperm roots (e.g. Bonacorsi and Seago, 2016; Brundrett and Tedersoo, 2020).

Therefore, the development and structure of endodermis in conifer leaves are both similar to and different from conifer roots. Conifer leaf endodermis is typically in State I, Casparian bands only. Where lignified, secondary cell wall thickening occurs, as in *Pinus resinosa* needles and *Pseudotsuga* petioles, it occurs only in the outer tangential cell wall and without suberin lamella formation. However, our findings also show that some outer tangential cell wall thickening in needles seen under epifluorescence occurs in these outer tangential walls in *Cedrus*, *Metasequoia*, *Larix* (deciduous species all), *Picea*, *Pseudotsuga* and most *Pinus* species, but these do not stain for lignin by the phloroglucinol test as they do in *Pinus resinosa* (Meicenheimer *et al*., 2008). However, other *Pinus* species, e.g. *P. radiata* (Donaldson and Williams, 2018) and *P. laricio* (Coulter and Chamberlain, 1901; Popham, 1966; Clegg and Cox, 1978), have what is like an innermost State III exodermis, except that there are no suberin lamellae and no U-shaped lignified walls. Interestingly, Griffith *et al*. (2014) showed a U-shaped wall in *Cycas hainanensis* leaf via phloroglucinol staining, but no suberin lamellae were demonstrated.

Age of needles must be very important to these differences (Soar, 1922; Cross, 1940; Ewers and Schmid, 1981; Sutinen *et al*., 2000; Wu *et al*., 2005; Sack and Scoffoni, 2013; Vasheka *et al*., 2019; Bozkurt *et al*., 2023; Huusk, 2024). In her recent thesis Huusk (2024) had bright-field images of phloroglucinol-stained *Pinus nigra*, *P. halapensis* and *P. pinea* in which needles older than first year in *P. nigra* appeared to reveal more heavily lignified cell walls under low magnification; yet, needles may remain on some pines like *P. sylvestris* for 3–4 years (Pensa and Jalkanen, 1999) and many *Picea* species retain needles for years. There would seem to be major physiological and ecological ramifications for leaves and the plants if leaf endodermis develops more lignified cell walls, even if only in outer tangential walls, over time as leaves survive and remain active, especially through seasons of warmth and then freezing cold, etc. Lastly, larger questions are: do Pinaceae represent earlier or later evolved gymnosperms (cf. Taylor *et al*., 2009; Lu *et al*., 2014; Gernandt *et al*., 2016; Yang *et al*., 2022) and thus when did endodermis evolve in gymnosperms and Pinaceae?
